# Osteoarthritis synovium as a nidus for monosodium urate crystal deposition inducing severe gout studied by label‐free stimulated Raman scattering combined with synovial organoids

**DOI:** 10.1002/mco2.70040

**Published:** 2025-01-05

**Authors:** Ziyi Chen, Wenjuan Wang, Yaxin Chen, Minbiao Ji, Yinghui Hu

**Affiliations:** ^1^ Department of Sports Medicine Huashan Hospital Fudan University Shanghai China; ^2^ State Key Laboratory of Surface Physics and Department of Physics Human Phenome Institute Multiscale Research Institute of Complex Systems Academy for Engineering and Technology Key Laboratory of Micro and Nano Photonic Structures (Ministry of Education) Fudan University Shanghai China

**Keywords:** gout, monosodium urate crystals, osteoarthritis, stimulated Raman scattering, synovial organoid

## Abstract

Gout, a common chronic disease, is characterized by the formation and deposition of monosodium urate (MSU) crystal deposition in articular and nonarticular structures. Osteoarthritis (OA), the most prevalent type of arthritis, is a progressive degenerative joint disease. Previous clinical studies have reported that gout frequently affects OA joints; however, the underlying mechanism remains unidentified. Recently, OA synovium has been proposed as a favorable vehicle for MSU crystal deposition. Therefore, this study aimed to investigate whether OA synovium acts as a nidus for MSU crystal deposition inducing severe gout flares, using label‐free, highly‐specific stimulated Raman scattering (SRS) microscopy combined with innovative preclinical models—synovial organoids. Crystal deposition, cellular phagocytosis, and subsequent inflammation intensity was imaged in ex vivo synovial organoids using SRS microscopy and other biochemical techniques. Results revealed that MSU crystals were more likely to deposit in OA synovium than in normal synovium. Furthermore, OA synoviocytes were more capable of phagocytosing crystals, leading to severe inflammation, and thus, expediting gout. These findings offer a potential explanation for why gout is preferred in OA joints and offer significant insights into the pathophysiology of gout, thereby informing prevention and management strategies for OA to prevent or alleviate the subsequent progression of gout.

## Graphical abstract

We investigated whether osteoarthritis (OA) synovium acts as a nidus for monosodium urate crystals deposition inducing severe gout using stimulated Raman scattering microscopy combined with synovial organoids. Crystal deposition, cellular phagocytosis, and subsequent inflammation was measured. Results revealed that crystals were more likely to deposit in OA synovium; OA synoviocytes were more capable of phagocytosing crystals; therefore, leading to severe inflammation, expediting gout

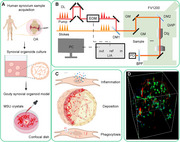

## 1 INTRODUCTION

Gout, a common chronic disease, is characterized by the formation and accumulation of monosodium urate (MSU) crystals within joints and other structures.^1^, ^2^ The prevalence and rate of impairment of gout have sharply grown recently, placing a significant financial burden on society.^3^, ^4^ Osteoarthritis (OA) is the most prevalent arthritis affecting all joint tissues, which can lead to pain, joint destruction, and handicap.^5^ Previous clinical studies have reported that gout frequently affects OA joints.^6^, ^7^, ^8^, ^9^, ^10^ An extensive case–control research involving nearly 80,000 individuals, indicated a strong correlation between OA and a higher risk of future gout development, boasting an odds ratio of 1.27.^11^ Additionally, a cross‐sectional study in community settings revealed a correlation between the sites of gout attacks and the existence of OA, indicating that OA‐affected joints may be at an increased risk of developing gout.^12^

We introduced the concept of “joint damage‐related events” as a contributor to gout development to identify the underlying mechanisms of why OA promotes gout.^13^ For example, our previous study showed that the release of collagen modified the crystal morphology and enhanced the initial immune inflammatory reactions trigged by MSU, thus aggravating gout.^14^ However, the specific roles of OA cells and tissues in the exacerbation of gout remain elusive. Observation of MSU crystals on the synovium via arthroscopy in clinical practice indicate that OA synovium may serve as a vehicle for MSU crystal deposits, thereby inducing severe gout.^10^, ^15^, ^16^ However, experimental validation is lacking.

With recent advancements in science and technology, novel biochemical and imaging techniques have been developed to address this gap. Stimulated Raman scattering (SRS) microscopy emerged as a cutting‐edge technique for chemical imaging in biomedical research renowned for its high imaging speed, chemical precision, superior spectral clarity, and three‐dimensional (3D) imaging capability.^17^, ^18^ We previously discovered that SRS microscopy can label‐freely identify MSU crystals in tissues, observe cellular phagocytosis of MSU crystals, and reflect tissue inflammatory infiltration based on distinctive fingerprint Raman spectra.^14^, ^19^ Organoids, as preclinical models of human diseases, allow for both in vivo and in vitro investigations and represent a recent innovation in modeling physiological processes of whole organisms.^20^ Synovial organoids have been successfully constructed^21^, ^22^ and used in disciplinary arthritis research.^21^, ^23^, ^24^ Leveraging the transparence of organoids, our preliminary study has successfully applied SRS to demonstrate the spatiotemporal distribution of MSU crystals in human synovial organoids.^25^

Therefore, this study aimed to explore whether and how OA synovium accelerates MSU crystal deposition and promotes gout, using organoid technology and SRS microscopy. Specifically, after successfully establishing synovial organoids, SRS microscopy combined with other biochemical techniques was used to investigate inflammation, crystal deposition, and cellular crystal phagocytosis in gout post‐OA. These findings offer a potential explanation for why gout preferentially affects OA joints and offer significant insights into the pathophysiology of gout, thereby informing prevention and management strategies for OA to prevent or alleviate the subsequent development of gout.

## 2 RESULTS

### 2.1 Gout post‐OA synovial organoid culture and validation

**FIGURE 1:**
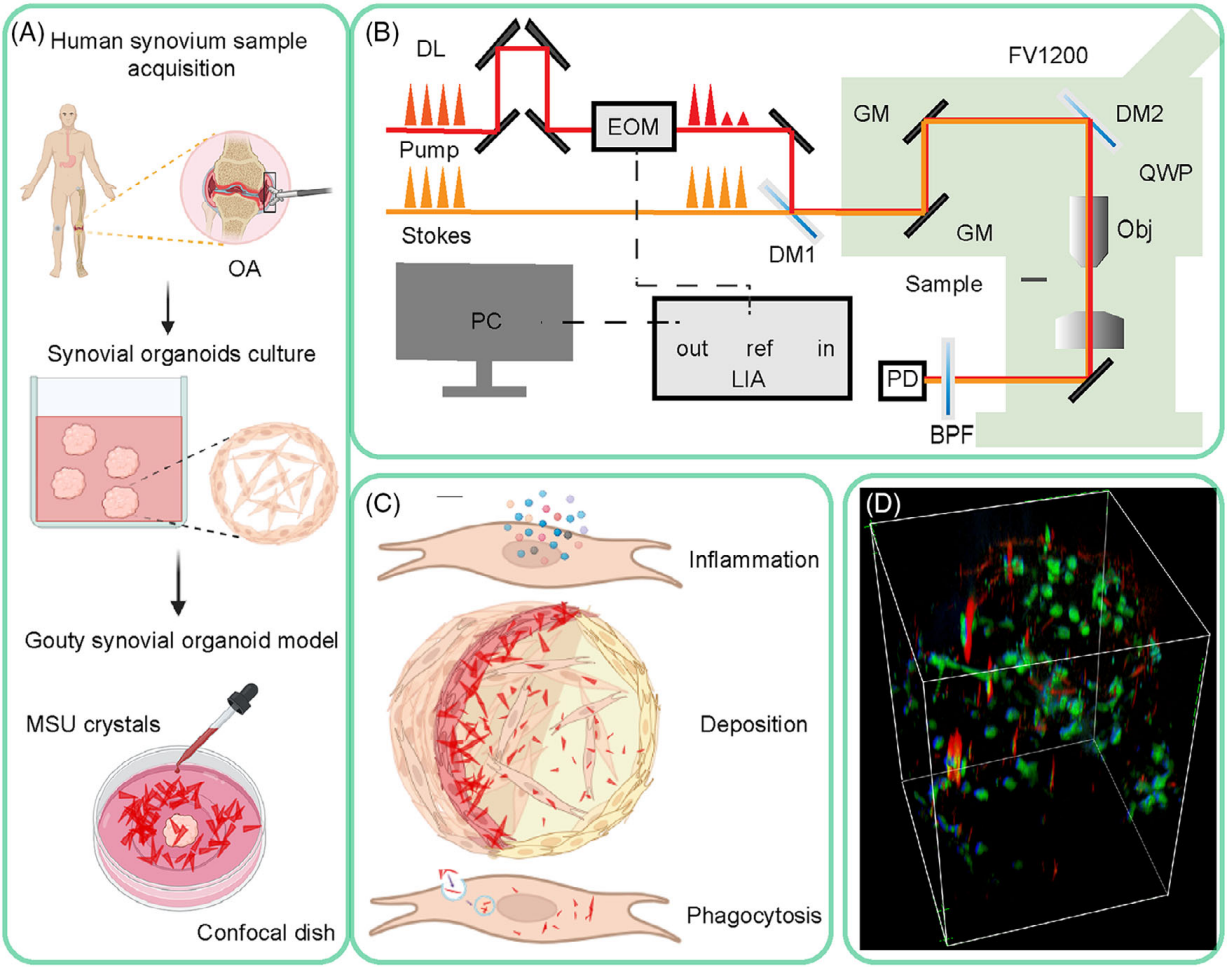
Illustration of the experimental design. (A) Organoid cultivation. Human synovial samples were obtained from patients with OA by arthroscopy. After digesting and seeding procedures, synovial organoids were formed after 21 days of culture. MSU crystals (50 mg/mL) were added to the synovial organoids. (B) Experimental setup. (C) Tissue inflammation, MSU crystal deposition, and cellular phagocytosis of crystals were analyzed using SRS imaging combined with other biochemical techniques. (D) 3D imaging of OA synovial organoids by SRS microscopy. MSU crystals (red, 630 cm^−1^), lipids (green, 2930 cm^−1^), and proteins (blue, 2930 cm^−1^). DL, delay line; EOM, electro‐optic modulator; DM, dichroic mirror; GM, galvo mirror; Obj, objective; BPF, bandpass filter; PD, photodiode; LIA, lock‐in amplifier.

Figure 1 depicts the overall design of the experiment, which shows 3D human synovial organoid model cultivation, SRS microscopy imaging, data collection, and quantification.

We first validated normal and OA synovial organoids. Figure S1A shows the synovial organoid cultivation procedures. Histological examination of the cultured synovial organoids revealed spontaneous accumulation and compaction of synoviocytes at the lining layer, exhibiting a remarkable anatomical resemblance to the human synovium.^26^ OA synovial organoids were thickened and arranged disorderly compared with normal synovial organoids (Figure S1B). In synovial organoids, synoviocytes autonomously generate extracellular matrix (ECM) components, such as reticular fibers, similar to those in human synovium.^26^, ^27^, ^28^ Gomori reticulin staining showed that the reticular fibers in the OA synovial tissues were more disorganized than those in the normal synovial organoids (Figure S1C). Lubricin, a key component of synovial fluid synthesized by synovial lining cells, is known to decrease in expression with cartilage degeneration or injury, as observed in animal models of acute joint trauma or OA.^29^ A reduction in immunoreactive lubricin in the lining layer of OA synovial organoids was confirmed (Figure S1D,G). To investigate inflammatory markers further, we conducted immunohistochemical staining for IL‐1βand TNF‐α, as depicted in Figure S1E,F. The findings revealed a markedly elevated expression of these inflammatory mediators in OA synovial organoids compared with normal controls (Figure S1G). Overall, OA synovial organoids were successfully established, showing more disordered tissue structure, less lubricin, and more inflammatory cytokine expression, similar to human OA synovium. MSU crystals were then added in vitro to organoids to generate gout post‐OA synovial organoids.

### 2.2 3D SRS imaging of gout post‐OA synovial organoids

**FIGURE 2:**
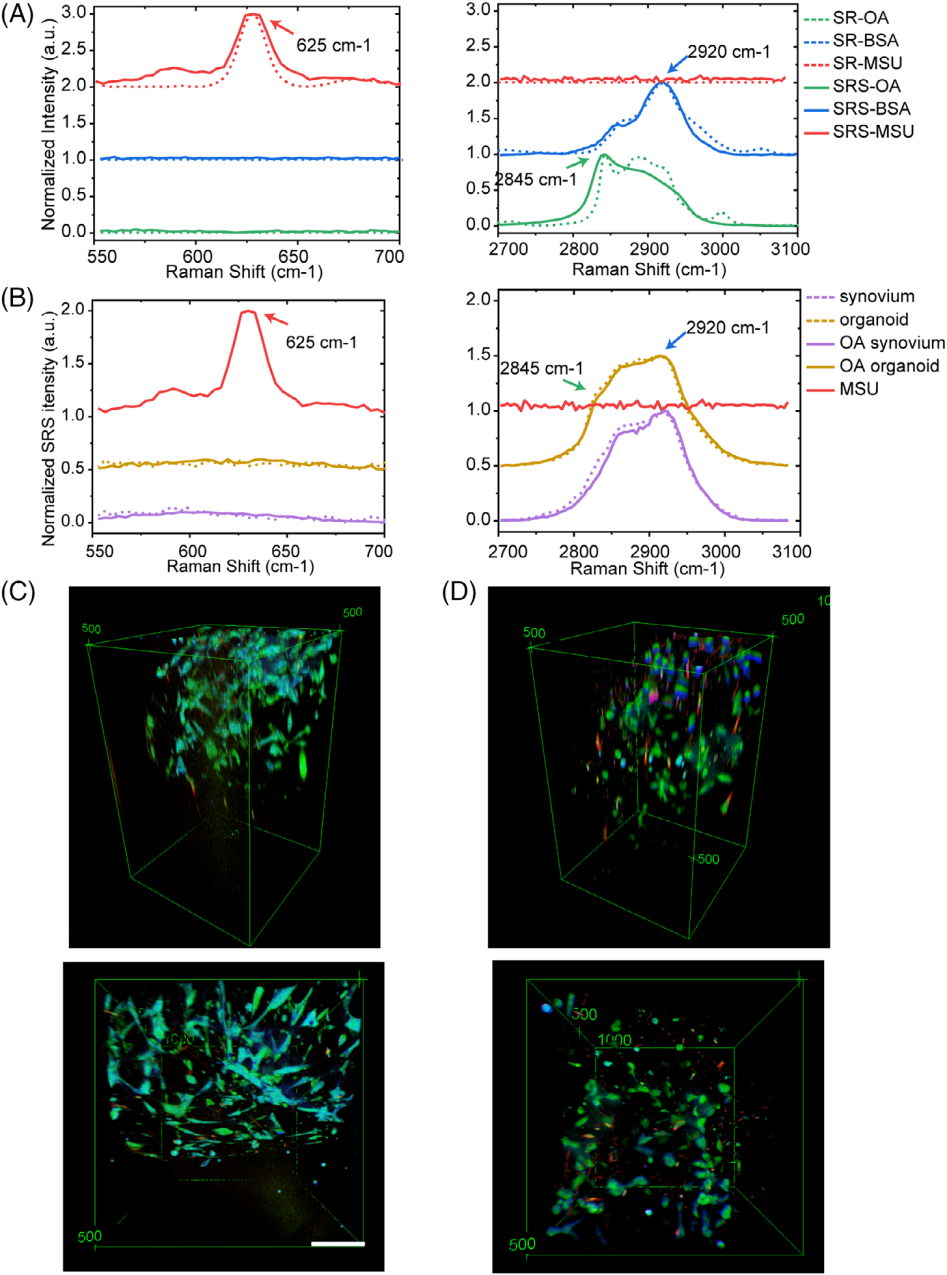
3D SRS imaging of gout post‐OA and gout synovial organoids. (A) Spontaneous and stimulated Raman spectral analysis of standard OA, bovine serum albumin (BSA), and MSU. (B) SRS spectra of MSU, normal synovial tissue, OA synovial tissue, normal synovial organoids, and OA synovial organoids. The synovial organoids showed similar SRS spectra to fresh synovial tissue. (C and D) Side view (upper panel) and top view (lower panel) of gout (C) and post‐OA (D) synovial organoids. MSU crystals (red, 630 cm^−1^), lipids (green, 2930 cm^−1^), and proteins (blue, 2930 cm^−1^). Scale bar: 100 µm.

Then 3D SRS imaging of synovial organoids was performed. Spontaneous Raman spectroscopy was performed in the range of 550–3100 cm^−1^ on standard chemicals to identify the spectral fingerprint of the experimental samples. The characteristic spectral patterns of the standard chemicals were successfully captured and reflected in the composite SRS spectrum. Raman spectral analysis revealed several distinct peaks, with notable intensifications observed at 625, 2920, and 2845 cm^−1^, corresponding to the vibrational modes of the purine ring in MSU crystals, CH3 stretching mode of proteins, and CH2 stretching mode of lipids, respectively (Figure 2A). The spectra of viable synovial organoids and fresh synovial tissues were consistent, indicating an effective organoid culture (Figure 2B). The no‐resonant (far from the vibration mode for imaging MSU crystals) control imaging is shown in Figure S2.

Using SRS microscopy, we visualized the 3D temporal characteristics of both normal and OA synovial organoids at intervals of 0, 4, 8, 12, 24, and 48 h poststimulation with MSU crystals (Figure S3). Figure 2C,D displays the comparative side and top views of normal and OA synovial organoids after an 8‐h exposure to MSU crystals.

### 2.3 Severe inflammation in gout post‐OA synovial organoids

**FIGURE 3:**
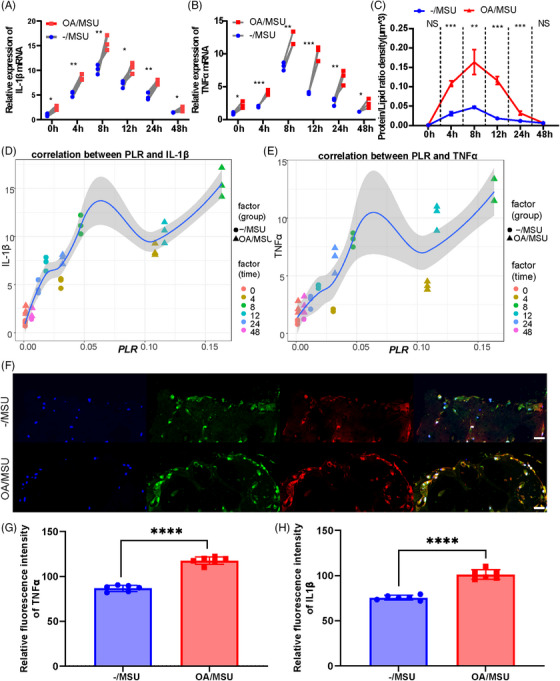
Severe inflammation in gout post‐OA synovial organoids. (A and B) Relative expression of IL‐1β (A) and TNF‐α (B) mRNA of normal and OA synovial organoids after adding MSU crystals for 0, 4, 8, 12, 24, and 48 h, respectively. (C) PLR density of normal and OA synovial organoids after adding MSU crystals for 0, 4, 8, 12, 24, and 48 h, respectively. (D and E) Correlation between PLR density and IL‐1β (D) and TNF‐α (E) mRNA expression. (F) Representative images of double immunofluorescence staining of IL‐1β and TNF‐α in normal and OA synovial organoids after adding MSU crystals for 8 h. Scale bar: 50 µm. (G and H) Relative fluorescence intensity of IL‐1β (G) and TNF‐α (H) in normal and OA synovial organoids after adding MSU crystals for 8 h. NS, not significant; *p < 0.05, **p < 0.01, and ***p < 0.001.

The levels of inflammatory mediators’ mRNA expression and the protein/lipid ratio (PLR) observed by Raman scattering at different time points were assessed to explore inflammatory responses in normal and OA synovial organoids after adding MSU crystals. Higher levels of inflammatory mediators were observed in gout post‐OA synovial organoids than in gout synovial organoids (Figure 3A,B). Maximal inflammatory responses occurred at 8 h after MSU stimulation, and this time point was chosen for subsequent murine experiments (Figure 3A,B). Furthermore, the PLR of the same synovial organoids after MSU stimulation for different times was detected by SRS microscopy to further validate the results. The results showed a significantly higher PLR of gout post‐OA synovial organoids than that of gout synovial organoids (Figure 3C). Correlation analysis of mRNA levels of inflammatory cytokines and PLR indicated strong correlations between their values, demonstrating good application of the PLR to reflect inflammatory reaction in this study (Figure 3D,E). Additionally, IL‐1β and TNF‐α immunofluorescence dual staining in gout and gout post‐OA synovial organoids was performed and imaged by laser confocal imaging (Figure 3F). The protein expression levels of inflammatory mediators were higher in gout post‐OA synovial organoids (Figure 3G,H). Therefore, the results showed that OA synovium augmented their sensitivity and mounted a hyper‐inflammatory response to secondary MSU stimuli, thus aggravating gout.

### 2.4 Enhanced MSU crystal deposition in gout post‐OA synovial organoids detected by SRS microscopy

**FIGURE 4:**
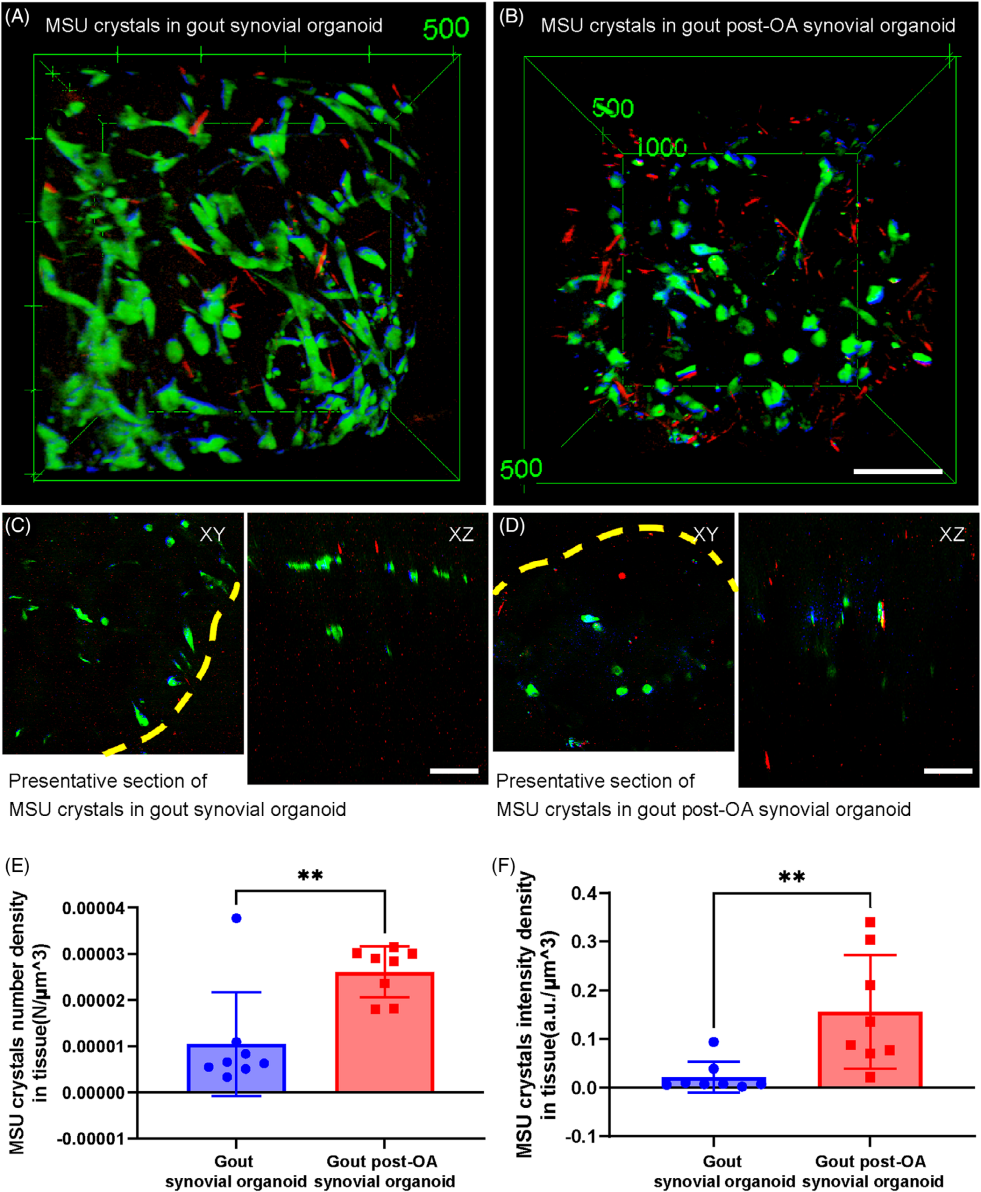
More MSU crystal deposition in gout post‐OA synovial organoids. (A and B) Stereographic top views of deposited MSU crystals in gout and gout post‐OA synovial organoids. (C and D) Presentative sectional images of deposited MSU crystals in gout (C) and gout post‐OA (D) synovial organoids. Yellow dashed lines show the surface of the organoids. (E and F) Number (E) and intensity (F) density of deposited MSU crystals in gout and gout post‐OA synovial organoids. MSU crystals (red, 630 cm^−1^), lipids (green, 2930 cm^−1^), and proteins (blue, 2930 cm^−1^). Scale bar: 100 µm. *p < 0.05 and **p < 0.01.

Spatial deposition of MSU crystals in organoids was analyzed to clarify the underlying causes of severe inflammation in gout post‐OA synovial organoids. Figure 4A shows the stereographic top views of normal and OA synovial organoids following an 8‐h exposure to MSU crystals. Figure 4B shows representative cross‐sectional images. Statistical analysis revealed that the number and intensity density of MSU crystals in gout post‐OA synovial organoids were higher than those in gout synovial organoids (Figure 4C,D). This finding indicated that MSU crystals were more likely to deposit in synovial organoids pre‐exposed to OA.

### 2.5 Stronger phagocytic ability of OA synoviocytes detected by SRS microscopy

**FIGURE 5:**
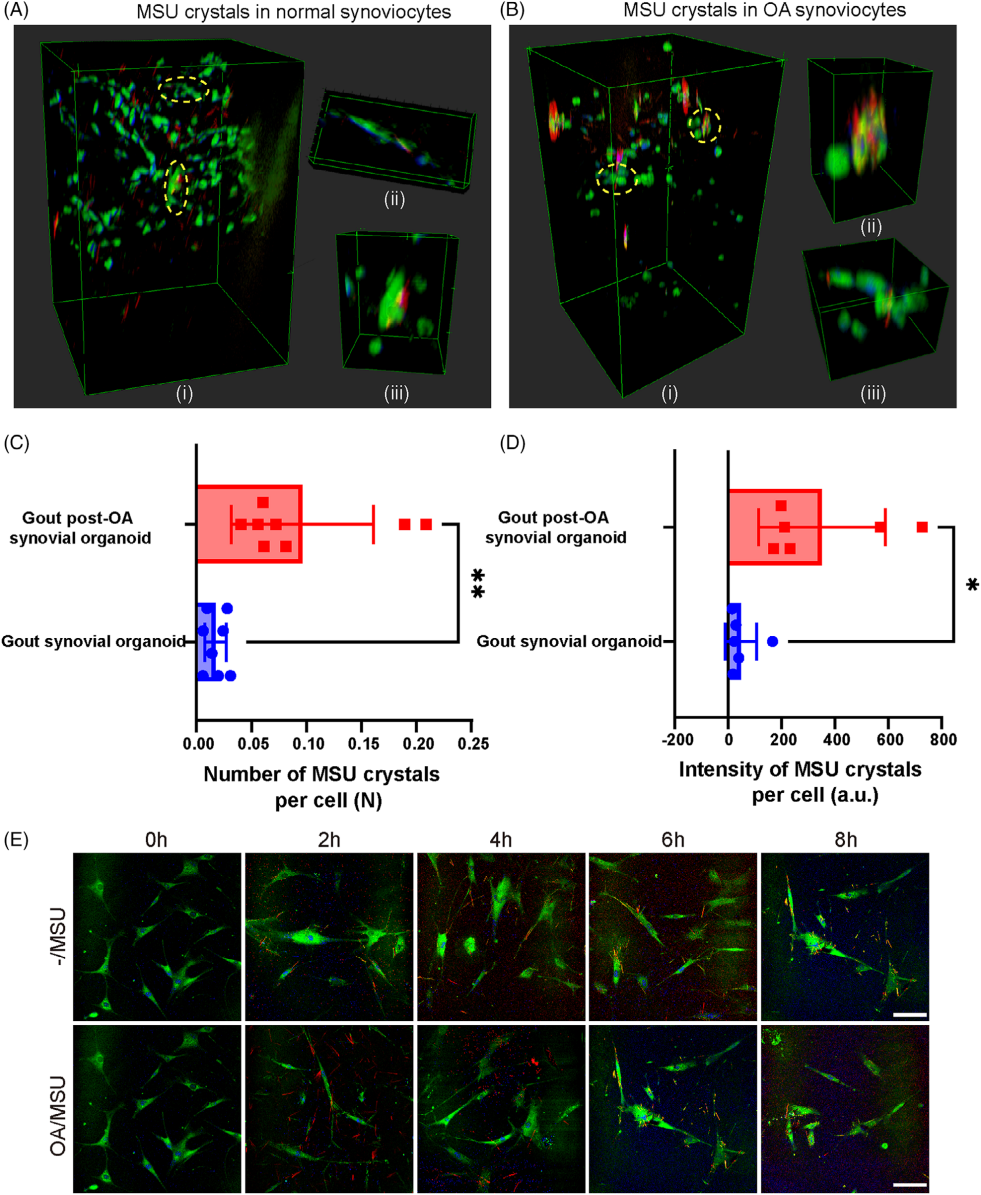
Stronger crystal phagocytosis ability of OA synoviocytes. (A and B) Stereographic side views (i) and representative zoom‐in images circled in yellow (ii, iii) of phagocyted MSU crystals in normal (A) and OA (B) synoviocytes in organoids. (C and D) Phagocytosed MSU crystal number (C) and intensity (D) per normal and OA synoviocytes in organoids. (E) SRS imaging of normal and OA synoviocytes stimulated by MSU crystals for 0, 2, 4, 6, and 8 h. Scale bar: 10 µm. (F) MSU crystal phagocytosis rate (percentage of intracellular MSU crystal number) in normal and OA synoviocytes. (G) MSU crystal phagocytosis rate (percentage of intracellular MSU crystal intensity) in normal and OA synoviocytes. MSU crystals (red, 630 cm^−1^), lipids (green, 2930 cm^−1^), and proteins (blue, 2930 cm^−1^). NS, not significant, *p < 0.05, **p < 0.01, and ***p < 0.001.

MSU crystals can be phagocytosed by synoviocytes. Therefore, we hypothesized that enhanced MSU crystal deposition could be attributed to stronger phagocytosis of these crystals. SRS was used to investigate how MSU crystals were phagocytosed and degraded after deposition in synovial organoids to identify the pathological process. 3D SRS stereoscopic side views and representative zoom‐in images of normal and OA synovial organoids after adding MSU crystals for 8 h were selected for better visualization of MSU crystals engulfed by synoviocytes (Figure 5A,B). Statistical analysis revealed that the number and intensity of MSU crystals in gout post‐OA synovial organoids were higher than those in gout synovial organoids (Figure 5C,D). Monolayer cell experiments of normal and OA synoviocytes stimulated with MSU crystals showed similar results (Figures 5E and S4A,B). This finding indicated that OA synoviocytes were more likely to phagocyte MSU crystals, possibly leading to more crystal deposition in tissues.

### 2.6 Inhibition of phagocytosis reduced inflammation in synoviocytes

**FIGURE 6:**
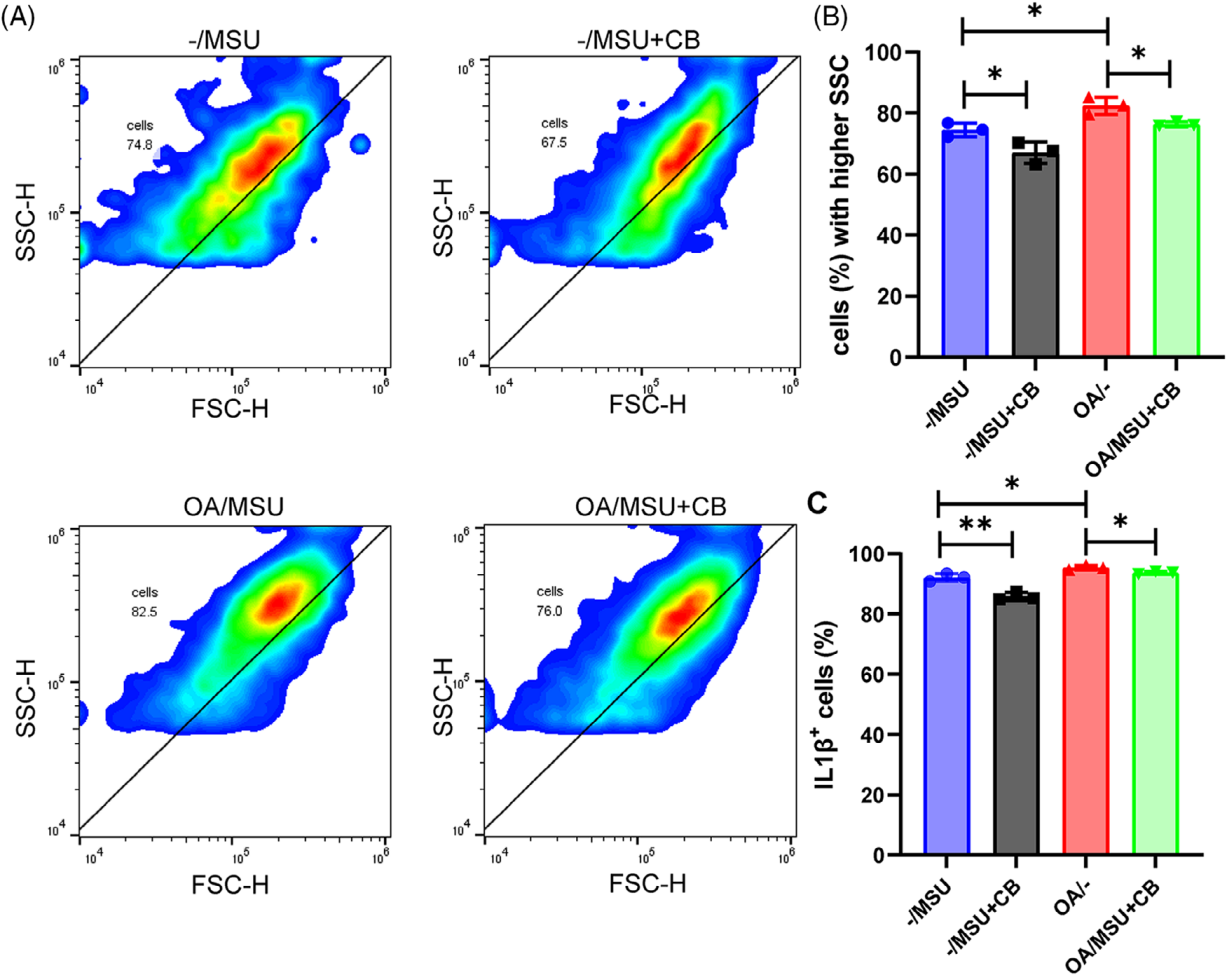
Inhibition of phagocytosis reduced inflammation in OA synoviocytes. (A and B) Flow cytometry of side scatter height (SSC‐H) of gout and gout post‐OA synovial organoids with or without the addition of CB. SSC‐H was applied to assess the level of phagocytosis by synoviocytes. (C and D) Percentage of IL‐1β+ synoviocytes in gout and gout post‐OA synovial organoids with or without adding cytochalasin B (CB).

Flow cytometric analyses of gout post‐OA and gout synovial organoids were performed (with or without the phagocytosis inhibitor cytochalasin B addition) to further explore the relationship between synoviocyte phagocytosis and synovial inflammation. Flow cytometric phagocytosis measurements showed that gout post‐OA synovial organoids were more capable of phagocytosis than gout synovial organoids (Figure 6A,B). Moreover, gout post‐OA synovial organoids showed higher IL‐1β levels (Figure 6C,D). As expected, the addition of cytochalasin B led to a notable decrease in phagocytosis capacity along with a subsequent reduction in IL‐1β levels. This finding indicated that OA synoviocytes displayed a stronger crystal phagocytosis ability, causing severe tissue inflammation.

### 2.7 MSU crystal deformation in gout post‐OA synovial organoids

Finally, to investigate MSU crystal deformation in synovial organoids, length of crystals was measured applying SRS imaging. The length of MSU crystals in tissues in gout post‐OA synovial organoids was shorter than that in gout synovial organoids (Figure S5A). Additionally, the length of MSU crystals phagocytosed by OA synoviocytes was shorter than that in normal synovial organoids (Figure S5B). The length of total MSU crystals and MSU crystals in tissues and cells gradually decreased in both synovial organoids (Figure S5C). These findings indicate that synovial organoids may degrade MSU crystals and that OA synovial organoids are more capable of processing MSU crystals.

## 3 DISCUSSION

Clinicians have long observed that individuals suffering from OA face an elevated likelihood of developing gout.^30^, ^31^ However, only a few studies have explored the underlying causes of this potential relationship.^32^ Thus, this study investigated whether the OA synovium serves as a nidus for MSU crystal deposition, inducing severe inflammation, using SRS microscopy combined with synovial organoid technology. This research provides the first demonstration that MSU crystals exhibit a greater propensity to deposit within OA synovial tissue, and that OA synoviocytes have a stronger ability to phagocyte crystals, leading to amplified inflammation and thus promoting the development of gout. These findings provide a potential explanation for why gout is preferred in OA joints and offer significant insights into the pathophysiology of gout, and indicate that effective intervention targeting the synovium at the OA stage may effectively slow down the progression of gout.

Synovium provides physical support for joints and lubricates joint surfaces.^33^, ^34^ However, recent studies have reported that the synovium in arthritis possesses the capacity to independently modulate the joint's inflammatory state rather than only acting as a “passive responder” to the inflammatory microenvironment.^35^ Activated synovial cells are reported to play a pivotal role in OA through the secretion of inflammatory mediators and matrix degradating enzymes that induce cartilage damage, thus worsening synovitis and forming a vicious cycle.^36^ Furthermore, the synovium plays an important role in gout, including secreting components that disrupt the equilibrium of urate levels^37^ and recruiting immune cells that amplify inflammation.^35^ Synoviocytes trigger inflammation through the phagocytosis of MSU crystals and escalate the release of pro‐inflammatory cytokines such as IL‐1β, IL‐6, IL8, TNF‐α, NLRP3, MCP‐1, as well as growth factors including NGF and HGF.^38^, ^39^ These discoveries underscore the significant role of the synovium in the progression of both OA and gout.

However, whether the OA synovium acts as a nidus for MSU crystal deposition remains largely unclear. Thus, this study aimed to clarify this with the use of preclinical models, organoids, and label‐free 3D imaging, SRS microscopy.^20^ The results showed that the OA synovium acted as a nidus for MSU crystal deposition, possibly caused by stronger phagocytosis of crystals by OA synoviocytes, thus leading to a hyper‐inflammatory response to a secondary MSU challenge. The pathological alternations in the OA synovium may be related to MSU crystal deposition and deformation. Fibrotic OA synoviocytes may overproduce ECM debris, such as fibronectin and type I collagen, predisposing them to MSU crystal formation.^31^, ^40^, ^41^ Furthermore, this study demonstrated that OA synovial organoids were more capable of MSU phagocytosis than normal synovial organoids. A number of factors have been identified to be correlated with MSU crystal phagocytosis, including ECM protein, cartilage particles, and inflammatory mediators generated during OA.^42^ Enhanced phagocytosis of MSU crystals can trigger more severe inflammatory cascades.^39^, ^43^, ^44^ These findings indicate that the OA synovium is a pathogenic nidus contributing to gout exacerbation.

Moreover, this study demonstrated that MSU crystals in gout post‐OA synovial organoids were smaller in length, indicating that OA organoids had a stronger ability to process MSU crystals. Several reasons could account for the phenomenon. First, cytokines and enzymes secreted by inflammatory synoviocytes degrade MSU crystals into shorter crystals.^42^, ^45^ We also discovered that collagen secreted by OA synovium leads to the formation of shorter MSU crystals.^31^ Additionally, previous studies have shown that crystals phagocytosed by synoviocytes may be digested and processed into smaller pieces.^38^, ^44^ Smaller crystals were more likely to penetrate the tissue, be deposited in it, and be engulfed by cells, forming a vicious cycle.^40^

For a long time, hyperuricemia has been considered as a necessary condition for developing gout. However, recent perspectives indicated that hyperuricemia alone is insufficient for the formation and deposition of crystals or for clinically manifested gout.^14^, ^46^, ^47^ Clinical research demonstrated that more than half of the individuals with serum urate exceeding 10 mg/dL do not experience gout over a 15‐year follow‐up period; conversely, some with serum urate levels below 6 mg/dL may still develop gout if other risk factors are present.^46^, ^47^ In clinical practice, we also observed that older female patients who are at high risk of OA with normal uric acid would also develop gout. Additionally, a radiographic study indicated that gout attacks often occur in OA joints.^47^ Therefore, OA synovium may serve as an intra‐articular risk factor for gout independent of hyperuricemia.

Limitations still reside in this study. First, we could not fully mimic the pathogenesis of gout patients, as synovial organoids were studied ex vivo. Furthermore, MSU crystals were added in vitro rather than spontaneous crystallization as in human joints. Therefore, a human 3D chip‐based chondro‐synovial coculture joint model^23^ and in vivo experiments are required to validate these findings. Furthermore, further researches are demanded to investigate the specific molecular mechanisms underlying the enhanced crystal phagocytosis by OA synoviocytes. Ultimately, this research did not encompass MSU deposition data from other types of organoids, like those of the aorta/arteries and kidneys, which could be a promising avenue for future investigations into the multi‐site nature of gout attacks.

Overall, our study offers clinical guidance for the prevention and treatment of gout by targeting the OA synovium. Patients with OA need prompt treatment to prevent the development of gout. Additionally, patients with high hyperuricemia should avoid OA to prevent gout flares. Furthermore, in individuals with both gout and OA, delaying the progression of OA while treating gout is important because the vicious cycle between the two diseases could aggravate joint damage and eventually lead to disability.

## 4 MATERIALS AND METHODS

### 4.1 MSU crystal preparation

The preparation of MSU crystals followed a previously established method.^45^ A total of 1 g of uric acid was dissolved in 200 mL of deionized water, and 450 mg of NaOH was introduced. This solution was then heated to 100°C, adjusted to pH 7.2 with HCl, and crystalize spontaneously at 4°C. Prior to each experimental use, the MSU crystals were sterilized by autoclaving. The crystals were then dried in an oven to achieve a consistent weight, after which the dried MSU crystals were weighed and reconstituted into a solution using Dulbecco's modified Eagle medium (DMEM) for culturing purposes.

### 4.2 Sample acquisition

Human OA synovial specimens were obtained from OA patients (n = 10, age 45–60 years, five males and five females) using the microfracture technique during arthroscopy. Non‐OA synovial tissues were collected from patients with meniscus or anterior cruciate ligament injuries who showed no signs of synovitis or cartilage injuries on arthroscopic examination (*n* = 10, age 45–60 years, five males and five females). Table S1 presents the basic characteristics of the surgical patients.

### 4.3 Synoviocyte culture

Synovial specimens were finely minced and enzymatically digested using 2 mg/mL collagenase type I (Sigma–Aldrich) in DMEM (Gibco). The obtained cell suspensions were then filtered through a 100‐um cell strainer (Gibco). These cells were subsequently seeded into cell culture flasks (Gibco) enriched with 10% fetal bovine serum (FBS; Gibco), along with penicillin and 1% streptomycin. The synoviocytes were allowed to grow and expand on the flask surface until they formed a confluent monolayer, under controlled conditions with a humidified atmosphere of 5% CO_2_ at 37°C. Postincubation and the treatment with the prepared MSU crystals, the culture medium was supplemented with 10 µg/mL of the phagocytosis inhibitor cytochalasin B (Sigma–Aldrich).

### 4.4 Organoid cultivation

Synovial organoids were cultivated as previously described.^26^ The well plates were coated with 1 mL/well of poly‐2‐hydroxyethylmethaacrylate (Sigma–Aldrich). Primary synoviocytes were then suspended in an ice‐cold Matrigel matrix (BD Biosciences) at a concentration of 1 × 10^6^ cells/mL. A drop of the Matrigel suspension was pipetted into each coated well. After 30 min of gelling, the Matrigel drops were overlaid with DMEM/F12 medium (Gibco), supplemented with 10% FBS and 1% penicillin–streptomycin (Gibco), and incubated in 5% CO_2_ at 37°C for 3 weeks. At various time points—0, 4, 8, 12, 24, and 48 h—MSU crystal solution was introduced to the synovial organoids to develop gout synovial organoids. The organoids underwent gentle washing to eliminate any surplus material, ensuring that only MSU crystals were retained within the organoids prior to examination under SRS microscopy. In total, 20 organoids were cultured (10 OA and 10 normal).

### 4.5 SRS experimental setup and data acquisition

SRS measurements were conducted utilizing a custom‐built system. A commercial femtosecond optical parametric oscillator, the Insight DS+ from Newport Inc., was employed to produce the source pump pulses (tunable 680–1300 nm, ∼150 fs) and Stokes beams (fixed at 1040 nm, ∼200 fs). To achieve hyperspectral SRS spectra, both femtosecond laser pulses were stretched to picosecond durations using glass rods, specifically to approximately 3.8 ps for the pump pulse and 1.8 ps for the Stokes beam. The combined pump and Stokes beams were directed into a laser scanning microscope (FV1200; Olympus), focused using an objective lens (UPLSAPO 25XWMP2, NA = 1.0; Olympus), and captured by an oil immersion condenser lens (NA = 1.4; Nikon). The stimulated Raman loss signal was detected using a photodiode with a bandpass filter (Chroma; ET890/220 M). The Stokes beam was modulated at approximately 20 MHz by an electro‐optical modulator (Thorlabs; EO‐AM‐R‐20‐C2), and the stimulated Raman loss signal was demodulated using a lock‐in amplifier (Zurich Instruments; HF2LI). A piezo actuator was used to vertically move the objective for 3D imaging capability. For imaging of the synovial organoids in this study, two specific pump–Stokes wavelength combinations were used: 802 + 1040 nm for lipid and protein on‐resonance to depict cells and 977 + 1040 nm for MSU on‐ and off‐resonance depicting MSU crystals. The crystal length varied between 3 and 30 µm in this study. SRS microscopy could identify tissues with minimal spatial resolution at the nanometer level. Most crystals could thus be identified both inside and outside the cells.

### 4.6 Processing of SRS image data

The data obtained from the SRS imaging were processed using ImageJ, an open‐source software platform, with 3D reconstruction performed for the synovial organoids. The process began with background subtraction for each chemical channel by subtracting the off‐resonant images. Then the organoid tissue and cell regions were segmented by creating masks to identify these areas. The regions corresponding to the organoids were manually delineated, whereas the cell regions were recognized by contrasting the background with the signal present in the lipid channel using the “Find Edges” function within ImageJ. The extracted areas were subsequently converted to binary masks. Nest, after selecting crystals with the “Threshold” function, and the “Analyze Particles” tool was used to calculate parameters indicative of crystals, including determining the length, aspect ratio, number, and intensity of MSU crystals within the image stacks, as well as those of the tissues and cells. Consistent intensity thresholds for MSU were maintained across different regions to ensure comparability. In a previous study, PLR was introduced as a new parameter to describe the intensity of immune cell recruitment and inflammatory cytokine secretion.^14^ Both the intensity density representing MSU crystals in units, and the PLR representing the level of tissue inflammation, were defined in this study.

$$\mathrm{PLR}=\frac{\;\mathrm{protein}\;\mathrm{intensity}}{\mathrm{lipid}\;\mathrm{intensity}}$$

$$\begin{array}{ccc} & & \mathrm{MSU}\;\mathrm{crystal}\;\mathrm{number}\;\mathrm{density}\;\mathrm{in}\;\mathrm{tissue}\;\;\left(\frac{N}{\mu m^{3}}\right)\\ & & \;=\;\frac{\;\sum \mathrm{MSU}\;\mathrm{number}}{\mathrm{areas}\;\mathrm{of}\;\mathrm{each}\;\mathrm{region}*\;\mathrm{vertical}\;\mathrm{spacing}}\\ & & \;\times \;\mathrm{MSU}\;\mathrm{intensity}\;\mathrm{density}\;\mathrm{in}\;\mathrm{tissue}\;\;\left(\frac{a.u.}{\mu m^{3}}\right)\\ & & \;=\;\frac{\;\sum \mathrm{MSU}\;\mathrm{intensity}}{\mathrm{areas}\;\mathrm{of}\;\mathrm{each}\;\mathrm{region}*\;\mathrm{vertical}\;\mathrm{spacing}} \end{array}$$

$$\begin{array}{ccc} & & M\mathrm{SU}\;\mathrm{crystal}\;\mathrm{phagocytosis}\;\mathrm{rate}\;\\ & & \;\left(\mathrm{percentage}\;\mathrm{of}\;\mathrm{intracellular}\;\mathrm{MSU}\;\mathrm{crystal}\;\mathrm{number}\right)\\ & & \;=\frac{\;\sum \mathrm{intracellular}\;\mathrm{MSU}\;\mathrm{number}}{\sum \mathrm{MSU}\;\mathrm{number}} \end{array}$$

$$\begin{array}{ccc} & & \mathrm{MSU}\;\mathrm{crystal}\;\mathrm{phagocytosis}\;\mathrm{rate}\;\\ & & \;\left(\mathrm{percentage}\;\mathrm{of}\;\mathrm{intracellular}\;\mathrm{MSU}\;\mathrm{crystal}\;\mathrm{intensity}\right)\\ & & \;=\frac{\;\sum \mathrm{intracellular}\;\mathrm{MSU}\;\mathrm{intensity}}{\sum \mathrm{MSU}\;\mathrm{intensity}} \end{array}$$

### 4.7 Histology and immunohistochemistry

All synovial organoids were harvested and fixed in a 10% buffered formalin. The fixed tissues were embedded in paraffin sectioned and subjected to hematoxylin and eosin (H&E) staining as well as reticulin staining. The H&E and reticulin stained sections were examined and scored by a pathologist in a blinded manner.

For immunohistochemistry, sections were first treated with 3% hydrogen peroxide to eliminate endogenous peroxidase activity and subsequently blocked with 1% bovine serum albumin (BSA; Sangon) to reduce nonspecific binding. The sections were incubated with primary antibodies specific to IL‐1β and TNF‐α, followed by the application of a biotinylated secondary antibody. Finally, the stained samples were visualized and photographed using a Zeiss Axio microscope.

### 4.8 Quantitative reverse transcription polymerase chain reaction

Total RNA was extracted from synovial organoids using TRIzol reagent (Invitrogen) and was reverse transcribed into complementary DNA (cDNA) with the aid of the PrimeScript RT Reagent Kit (Takara). For real‐time quantitative reverse transcription polymerase chain reaction (, a reaction mixture was prepared consisting of 2 × SYBR Master Mix (5 µL), each primer (0.25 µL), and diluted cDNA (4.5 µL), resulting in a total reaction volume of 10 µL per well. The mixture was loaded into a 96‐well plate, with duplicates for each sample. Glyceraldehyde 3‐phosphate dehydrogenase served as an endogenous control for normalization. Relative quantification of target gene expression was determined employing the 2^−ΔΔCT^ method, a standard approach for analyzing gene expression data. The primer sequences used in this study were as follows: IL‐1β forward, 5′‐GGCGGCATCCAGCTACGAATC‐3′; IL‐1β reverse, 5′‐GAAGGGAAAGAAGGTGCTCAGGTC‐3′; TNF‐α forward, 5′‐CAATGGCGTGGAGCTGAGAGATAAC‐3′; TNF‐α reverse, 5′‐TCTGGTAGGAGACGGCGATGC‐3′.

### 4.9 Immunofluorescence assay

Synovial organoids were fixed in a 4% paraformaldehyde and then permeabilized with 0.1% Triton X‐100. After permeabilization, the organoids were thoroughly washed three times with phosphate‐buffered saline (PBS) to remove any residual Triton X‐100.A blocking step with 1% BSA was then performed for 1 h to minimize nonspecific antibody binding. The organoids were then incubated overnight at 4°C with primary antibodies specific to IL‐1β and TNF‐αovernight. On the subsequent day, the organoids were incubated with a Cy3‐conjugated secondary antibody (Invitrogen) for 1 h at room temperature. After washing with PBS, the nuclei were counterstained with 4′,6‐diamidino‐2‐phenylindole reagent. Finally, the prepared sections were visualized using a confocal laser fluorescence microscope (Olympus), and the images were analyzed and quantified using ImageJ.

### 4.10 Flow cytometry

The anti‐IL‐1β antibody (Abcam) was used in the following experiments. Gout post‐OA and gout synovial organoids were digested for cells and suspended in PBS for 30 min at 4°C, followed by a single wash and resuspension in PBS. Unstained cells were used as controls. Cell acquisition and analysis were performed using a FACS Calibur (BD Biosciences) with CellQuest Pro software (BD Biosciences). Flow cytometry data were analyzed utilizing FlowJo v10.8.0 software.

### 4.11 Statistical analysis

All calculations and graphical representations were carried out using GraphPad Prism version 9.0.0. Data are presented as mean ± standard deviation. To assess the significance of differences between groups, a Student's *t*‐test was applied, with *p* values < 0.05 indicating statistical significance.

## AUTHOR CONTRIBUTIONS

Yinghui Hua and Minbiao Ji proposed the concept of this research. Ziyi Chen and Wenjuan Wang performed the biochemical and cellular experiments. Yaxin Chen conducted the SRS microscopy experiments. Ziyi Chen, Wenjuan Wang, and Yaxin Chen contributed equally to data analysis, initial manuscript drafting, and manuscript refinement. Prior to submission, Minbiao Ji and Yinghui Hua examined and corrected the article critically. The final manuscript was reviewed and approved by all the authors.

## CONFLICT OF INTEREST STATEMENT

No conflict of interest was disclosed by any of the authors.

## ETHICS STATEMENT

This study was approved by the Ethics Committee of Huashan Hospital (KY2023‐807) and conducted in accordance with the guidelines of the 1975 Declaration of Helsinki with all participants signing an informed consent form.

## Supporting information



Supporting Information

## Data Availability

All data are available in the main text or in the supporting information.
