# Supplementary material for: Osteoarthritis synovium as a nidus for monosodium urate crystal deposition inducing severe gout studied by label‐free stimulated Raman scattering combined with synovial organoids
Source: MedComm (2020). 2025 Jan 5;6(1):e70040. doi: 10.1002/mco2.70040 (PMC11702473; doi:10.1002/mco2.70040)
Supplement: Supplementary file 1 — Supporting Information [file MCO2-6-e70040-s001.docx]

**Osteoarthritic Synovium as a Nidus for Urate Crystals Deposition Inducing Severe Gout Attack Studied by Label-Free Stimulated Raman Scattering Combined with Synovial Organoids**

Short title: Osteoarthritic Synovium Nidus for Urate Crystals

Ziyi Chen^1#^, Wenjuan Wang^1，3#^, Yaxin Chen^2#^, Minbiao Ji^2*^, and Yinghui Hua^1*^

^1^Department of Sports Medicine, Huashan Hospital, Fudan University, Shanghai, China

^2^State Key Laboratory of Surface Physics and Department of Physics, Human Phenome Institute, Multiscale Research Institute of Complex Systems, Academy for Engineering and Technology, Key Laboratory of Micro and Nano Photonic Structures (Ministry of Education), Fudan University, Shanghai 200433, China

^3^Shenzhen Sixth People's Hospital (Nanshan Hospital), Huazhong University of Science and Technology Union Shenzhen Hospital, Shenzhen 518052, China.

*Address correspondence to: Yinghui Hua; hua_cosm@aliyun.com and Minbiao Ji; minbiaoj@fudan.edu.cn

^#^These authors contributed equally to this work.

**Figure S1.** Normal and OA synovial organoid culture and validation. (A)Synovial organoid culture and engineering platforms. (B-C) The representative images of HE (B), Gomori reticulin (C) staining of human normal (left panel) and OA (right panel) synovial organoids. (D-F) The representative images of IHC staining of lubricin (D), IL-1β(E) and TNF-α(F) in normal (left panel) and OA (right panel) synovial organoids. (G) The relative expression of lubricin (left panel), IL-1β(middle panel) and TNF-α(right panel) positive cells in human normal and OA synovial organoids. For all figures: ** represents p < 0.01. Scare bar: 20um.


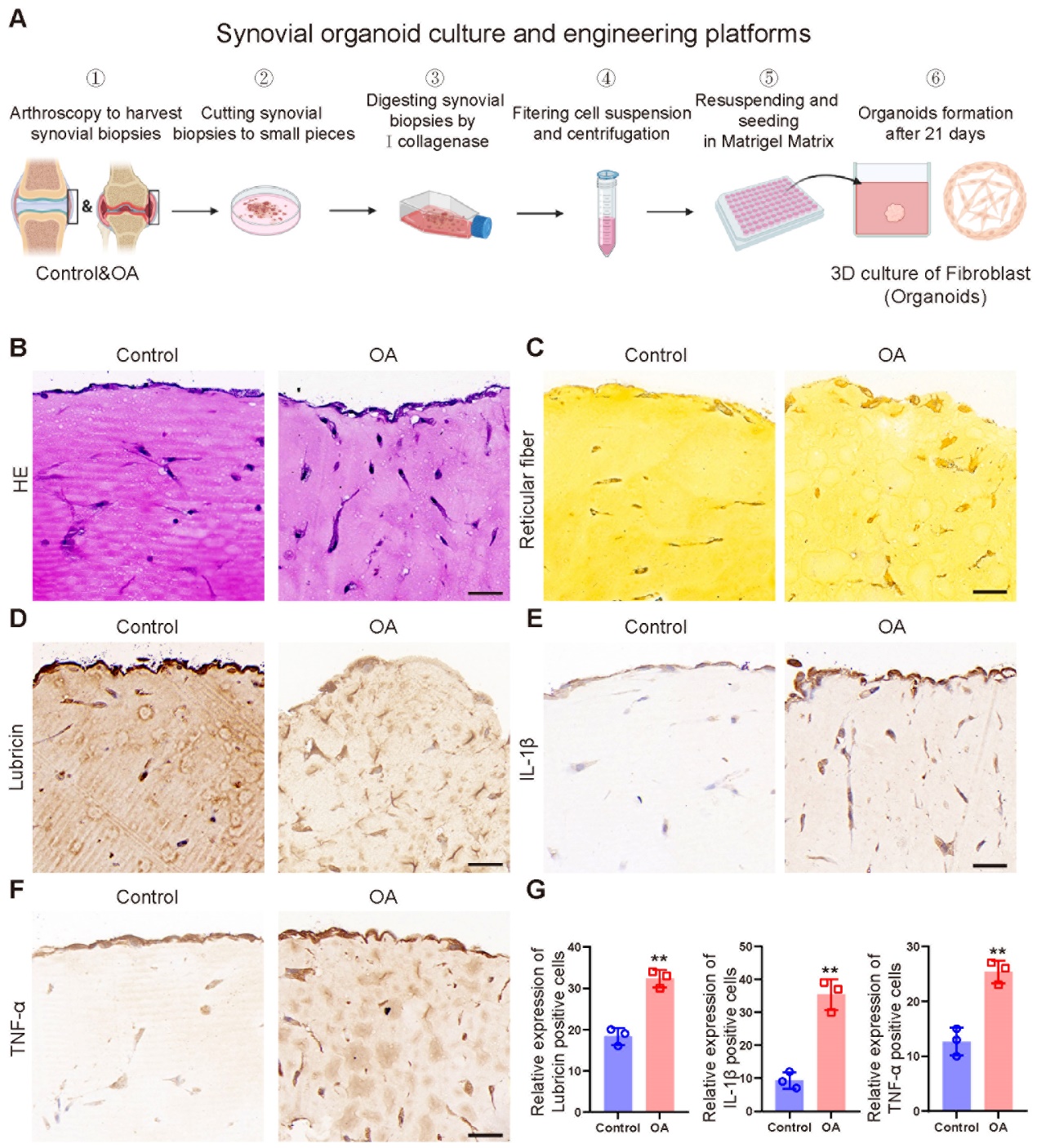


**Figure S2.** The on-resonance and off-resonance imaging of standard MSU and MSU in organoid.


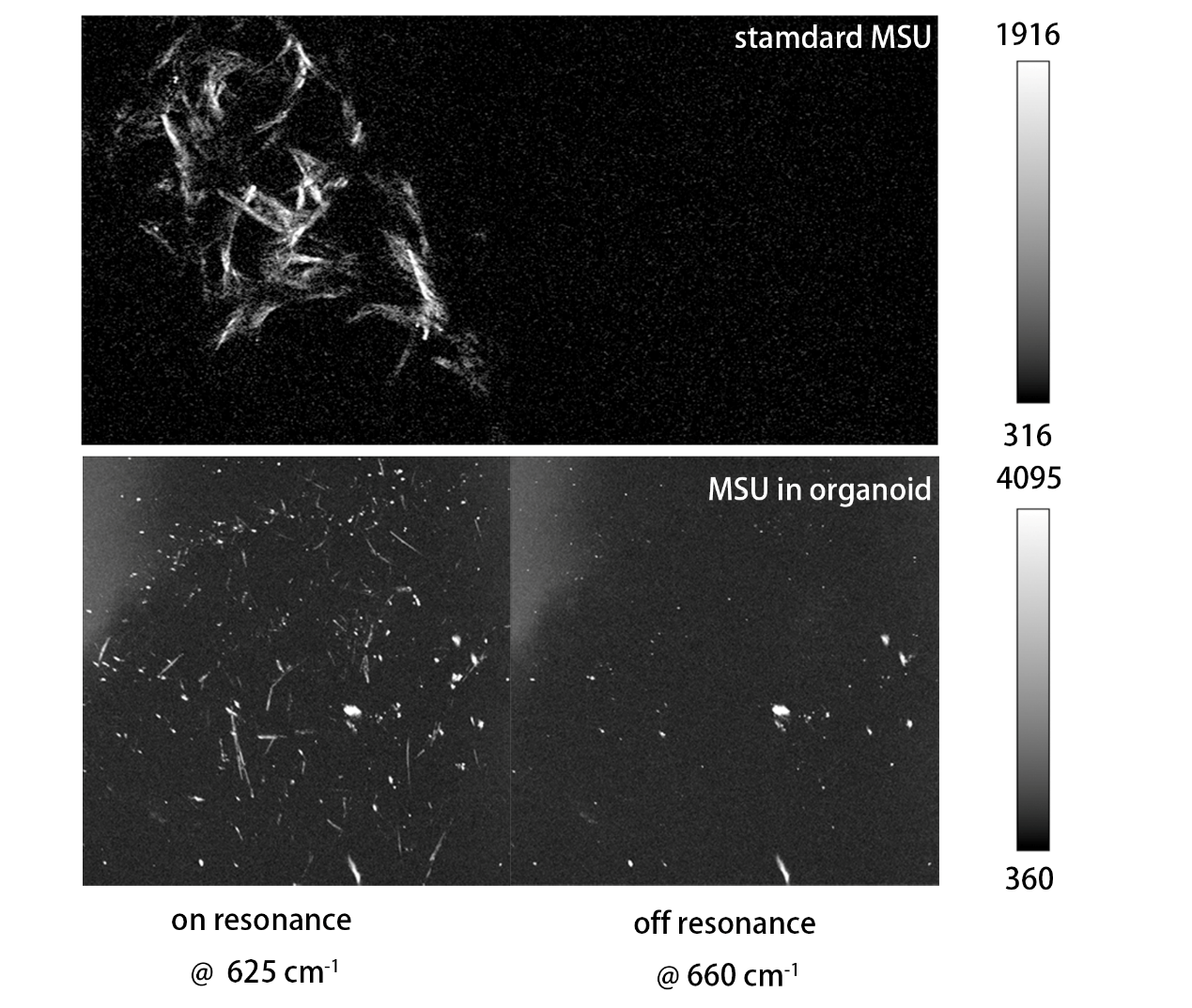


**Figure S3.** 3D time series SRS images of normal and OA synovial organoids stimulated by MSU crystals. Representative stereographic side views of human normal (A) and OA (B) synovial organoids after adding MSU crystals for 0, 4, 8, 12, 24 and 48 hours. MSU crystals (red, 630 cm-1), lipid (green, 2930 cm-1) and protein (blue, 2930 cm-1).


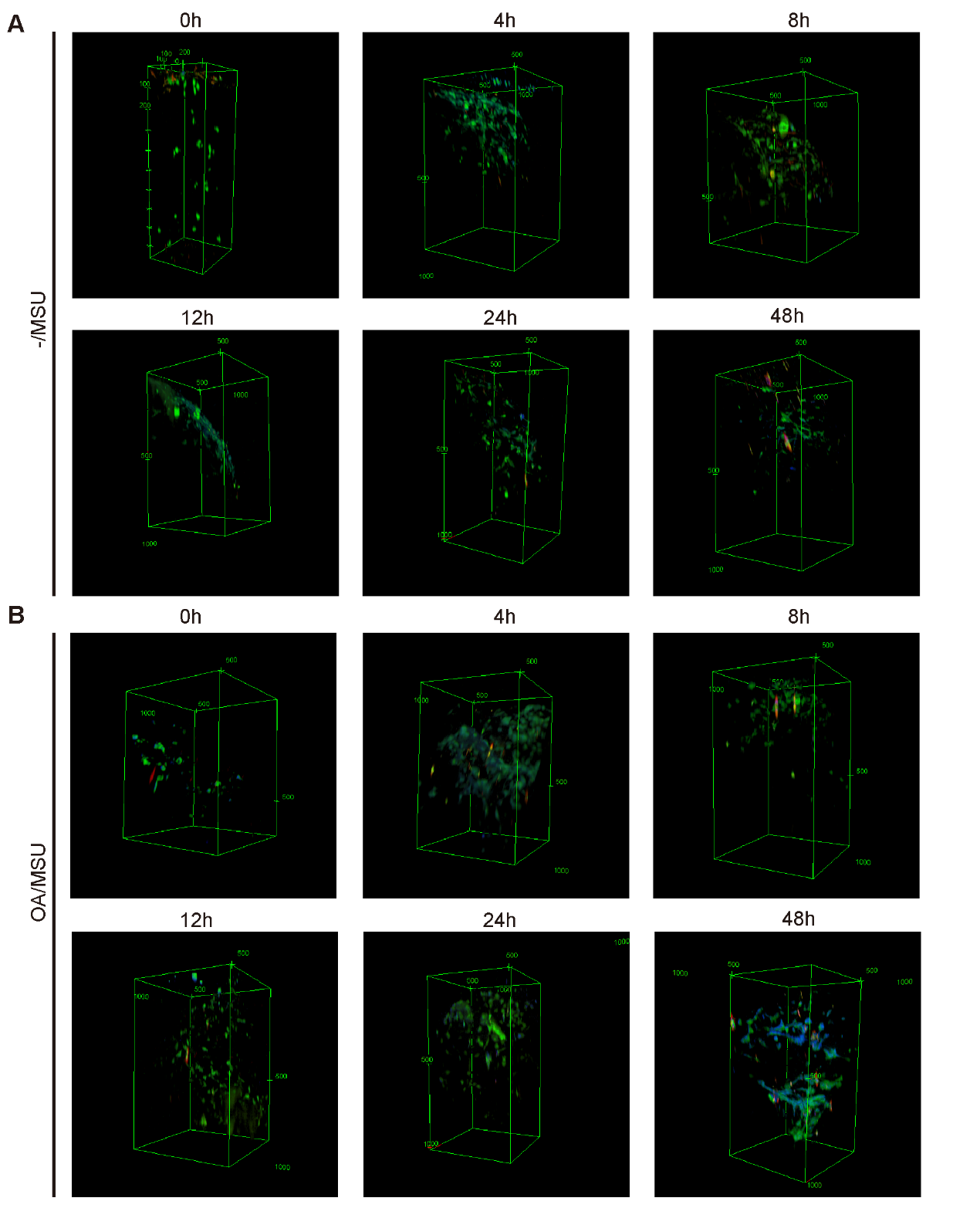


**Figure S4.** MSU crystals deformation. (A) Length of total MSU crystals in normal and OA synovial organoids system. (B) Length of MSU crystals in normal and OA synovial organoids tissues. (C) Length of MSU crystals in normal and OA synoviocytes. (D) MSU crystals in total, tissue and cells. For all figures: NS, not significance, * represents p < 0.05, ** represents p < 0.01, *** represents p < 0.001, **** represents p < 0.0001.


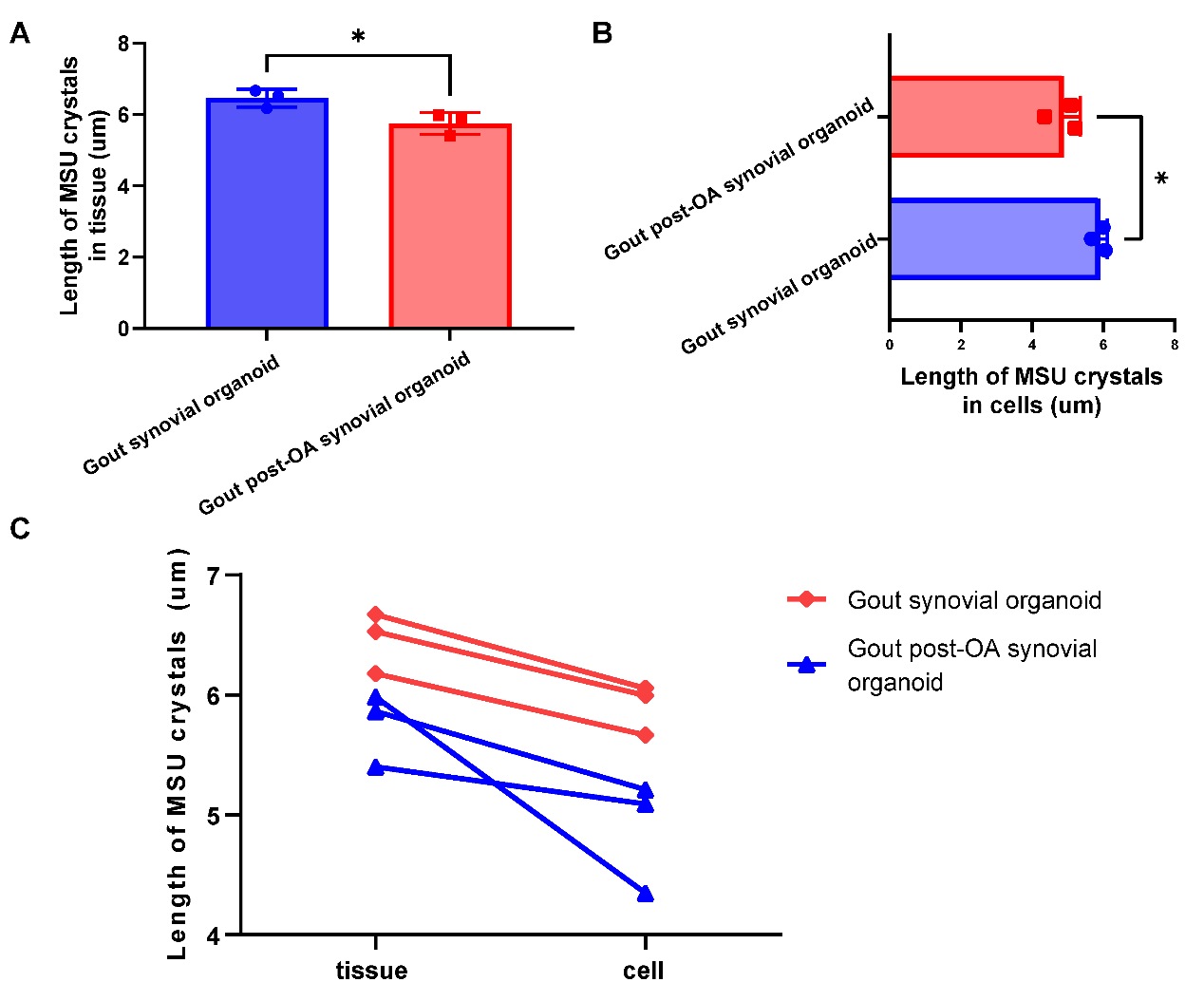


**Table S1 Basic characteristics of surgical patients**

| Year (y) | Gender | Diagnosis | Uric acid concentration (mmol/L) |
| --- | --- | --- | --- |
| 52 | female | Rupture of the anterior cruciate ligament of the right knee | 0.221 |
| 54 | male | Rupture of the anterior cruciate ligament of the right knee | 0.298 |
| 55 | female | Meniscus injury of the right knee | 0.217 |
| 49 | female | Rupture of the anterior cruciate ligament of the right knee | 0.331 |
| 60 | male | Meniscus injury of the right knee | 0.281 |
| 57 | male | Meniscus injury of the right knee | 0.329 |
| 60 | female | Rupture of the anterior cruciate ligament of the right knee | 0.297 |
| 48 | male | Rupture of the anterior cruciate ligament of the right knee | 0.378 |
| 52 | female | Meniscus injury of the right knee | 0.352 |
| 47 | male | Rupture of the anterior cruciate ligament of the right knee | 0.263 |
| 55 | female | Osteoarthritis | 0.238 |
| 52 | male | Osteoarthritis | 0.308 |
| 59 | female | Osteoarthritis | 0.279 |
| 49 | female | Osteoarthritis | 0.306 |
| 55 | male | Osteoarthritis | 0.251 |
| 56 | male | Osteoarthritis | 0.309 |
| 50 | female | Osteoarthritis | 0.260 |
| 46 | male | Osteoarthritis | 0.308 |
| 52 | female | Osteoarthritis | 0.274 |
| 49 | male | Osteoarthritis | 0.290 |
